# Using Extracellular Vesicles Released by GDNF-Transfected Macrophages for Therapy of Parkinson Disease

**DOI:** 10.3390/cells11121933

**Published:** 2022-06-15

**Authors:** Yuling Zhao, Matthew J. Haney, John K. Fallon, Myosotys Rodriguez, Carson J. Swain, Camryn J. Arzt, Philip C. Smith, Matthew Shane Loop, Emily B. Harrison, Nazira El-Hage, Elena V. Batrakova

**Affiliations:** 1 Center for Nanotechnology in Drug Delivery, University of North Carolina at Chapel Hill, Chapel Hill, NC 27599, USA; yulingz@email.unc.edu (Y.Z.); mjhaney@email.unc.edu (M.J.H.); 2 Eshelman School of Pharmacy, University of North Carolina at Chapel Hill, Chapel Hill, NC 27599, USA; jfallon@email.unc.edu (J.K.F.); cswain@unc.edu (C.J.S.); camiarzt@live.unc.edu (C.J.A.); pcs@email.unc.edu (P.C.S.); mloop@email.unc.edu (M.S.L.); emilybrookeharrison@gmail.com (E.B.H.); 3 Herbert Wertheim College of Medicine, Florida International University, Miami, FL 33199, USA; myrodrig@fiu.edu (M.R.); nelhage@fiu.edu (N.E.-H.)

**Keywords:** drug delivery, extracellular vesicles, GDNF, intranasal administration, neuroinflammation, Parkinson disease

## Abstract

Extracellular vesicles (EVs) are cell-derived nanoparticles that facilitate transport of proteins, lipids, and genetic material, playing important roles in intracellular communication. They have remarkable potential as non-toxic and non-immunogenic nanocarriers for drug delivery to unreachable organs and tissues, in particular, the central nervous system (CNS). Herein, we developed a novel platform based on macrophage-derived EVs to treat Parkinson disease (PD). Specifically, we evaluated the therapeutic potential of EVs secreted by autologous macrophages that were transfected ex vivo to express glial-cell-line-derived neurotrophic factor (GDNF). EV-GDNF were collected from conditioned media of GDNF-transfected macrophages and characterized for GDNF content, size, charge, and expression of EV-specific proteins. The data revealed that, along with the encoded neurotrophic factor, EVs released by pre-transfected macrophages carry GDNF-encoding DNA. Four-month-old transgenic Parkin Q311(X)A mice were treated with EV-GDNF via intranasal administration, and the effect of this therapeutic intervention on locomotor functions was assessed over a year. Significant improvements in mobility, increases in neuronal survival, and decreases in neuroinflammation were found in PD mice treated with EV-GDNF. No offsite toxicity caused by EV-GDNF administration was detected. Overall, an EV-based approach can provide a versatile and potent therapeutic intervention for PD.

## 1. Introduction

The National Parkinson Foundation^®^ estimates that Parkinson disease (PD) affects more than 1 million individuals in the U.S., with up to 60,000 new cases diagnosed each year. One of the greatest challenges is to provide efficient treatment for PD besides the replacement of neurotransmitters. Loss of dopaminergic neurons in the Substantia Nigra pars compacta (SNpc), along with inflammation in the brain and production of an excessive amount of reactive oxygen species (ROS), is a hallmark of PD. While many potent therapeutic proteins, including antioxidants and neurotrophic factors, were identified, the blood–brain barrier (BBB) remains a seemingly insurmountable obstacle to the routine use of systemically administered macromolecules. Contrary to stroke [1], diabetes [2], or traumatic brain injury [3], which can severely disrupt the BBB, PD patients usually do not have substantial changes in BBB permeability. Nevertheless, later advances indicate that some BBB disfunction may be developed in the late stages of neurodegenerative disorders, including PD dementia [2].

Different strategies are currently being developed to improve drug delivery across the BBB. They can be divided into two principal groups: increasing the drug influx into the brain and restricting the drug efflux out of the brain. Approaches for the first group include: (i) modifying a drug’s chemical structure, for example, increasing the lipophilicity of the molecule [4]; (ii) using carrier-mediated and receptor-mediated transcytosis [5]; or (iii) incorporating a drug into micro- and nanocontainers, including EVs [6]. The second group, which is focused on restricting drug efflux, includes co-administrating competitive or non-competitive inhibitors of drug efflux transporters, such as P-glycoprotein (Pgp), multidrug resistance protein (MRP), breast cancer resistance protein (BCRP), multi-specific organic anion transporter (MOAT), the members of the ATP-binding cassette (ABC) family [5,7].

In this work, we utilized a strategy that belongs to the first group: using EVs for the transport of a potent neurotrophic factor, GDNF, and we have provided proof-of-concept in a transgenic mouse PD model, Parkin-Q311X(A) mice. GDNF is known to promote neuronal survival, which is essential for mitigating neurodegeneration in PD patients [8,9,10,11,12,13]. Furthermore, a restoration and regeneration of dopaminergic neurons by activated macrophages and microglia that overexpress GDNF was demonstrated earlier during the natural healing process in injured striatum [14]. Regrettably, current attempts to deliver GDNF to the CNS have been hampered by pharmacological issues, including poor penetration across the BBB and offsite toxicity upon systemic administration. Thus, in recent clinical investigations, administration of GDNF alone failed to demonstrate clinical efficacy in PD patients [15]. However, these brain infusions carry a high risk of adverse effects and have poor patient adherence. As such, the development of innovative strategies that allow efficient GDNF delivery to the brain is of great importance.

In this regard, the field of nanotechnology holds enormous promise for the development of versatile drug-delivery systems. The incorporation of drugs into nanocarriers protects them against degradation and elimination from the bloodstream and allows for the targeting of therapeutics to a disease site. Much effort has been dedicated to the development of nanoformulations for drug delivery [16,17], but these efforts have been met with limited success. In fact, the opsonization of drug-loaded synthetic nanoparticles in the bloodstream has caused two main problems, nanotoxicity and rapid drug clearance by the mononuclear phagocyte system (MPS). To circumvent this problem, we engineered an EV-based, biomimetic delivery system capable of targeted transport of this neurotrophic factor to the brain. Using EVs as beneficial bio-nanostructures has the potential to overcome the major drawbacks related to opsonization and cytotoxicity and to introduce them for clinical application. Composed of cellular membranes with multiple adhesive proteins on their surfaces, EVs are known to specialize in cell–cell communication, facilitating the transport of their cargo to target cells. It was reported that EVs penetrate the BBB and can deliver incorporated therapeutics upon systemic and local administration [18,19,20,21]. Three different mechanisms for EV transport across the BBB have been suggested: receptor-mediated transcytosis, lipid-raft-mediated endocytosis, and macropinocytosis [20]. In addition, EVs exert unique biological activity, reflective of their origin, which allow for the capitalization on specific properties of parent cells and the amplification of the therapeutic effects of EV-based formulations [22]. These exceptional features of EVs should work in concert to dramatically improve the therapeutic efficacy of current treatment strategies utilizing GDNF.

In our earlier investigations, we assessed several types of parent cells and selected inflammatory response cells, macrophages that allow for targeted drug delivery to the inflamed brain in PD animals. Of note, using macrophages as parent cells is crucial for targeting EVs to inflamed brain tissues. We reported recently that EVs released by macrophages can accumulate in inflamed brain tissues in PD mice at greater amounts than those released by neurons or astrocytes [23]. Specifically, we demonstrated the remarkable abilities of immunocyte-derived EVs to interact with recipient cells [24,25,26], target inflamed brain tissues via LFA1/ICAM1 interactions, and deliver their therapeutic cargo [22,27,28], resulting in profound therapeutic effects in mice with acute neuro-inflammation induced by lipopolysaccharide (LPS), 6-hydroxydopamine (6-OHDA), or 1-methyl-4-phenyl tetrahydropyridine (MPTP). However, these toxin-induced PD models resemble PD at late stages, whereas transgenic animal models are more appropriate for representing early stages of the disease. In this work, we used Parkin-Q311(X)A mice, a transgenic model of PD with a mutation that produces C-terminally truncated parkin. These mice slowly develop the degeneration of dopaminergic neurons and progressively decrease in motor activity levels over several months [29].

EVs have already been recognized as promising drug nanocarriers for clinical use. Currently, over 20 clinical trials involving EVs can be found at http://www.clinicaltrials.gov (accessed on 2 March 2022). Most of them, especially those using mesenchymal stem cell (MSCs)-derived EVs, showed high feasibility, significant enhancement of antitumor immune response, and no safety concerns [30,31,32,33,34]. However, there are some drawbacks, including the upscaling processes of isolation and purification, as well as the efficient loading of these natural nanovesicles with various therapeutics. Concerning the manufacture of large lots of EVs nanocarriers, we reported earlier that EV formulations can be lyophilized and then restored without altering their morphology [24,25]. More information can be found in the recent review [28].

Regarding methods of drug incorporation into EV nanocarriers, we earlier developed exogenous-loading, naïve EVs isolated from parent-cell-conditioned media. Different techniques were used for loading therapeutics into EVs, including co-incubation, freeze-thaw cycles, sonication, electroporation, extrusion, and permeabilization of EV membranes with saponin [19,21,25,26,35,36,37]. As a result, EV-based formulations with high loading efficacy, sustained drug release, and preservation against degradation and clearance were manufactured. In the present work, we utilized another approach: the endogenous loading of EVs with GDNF, which was accomplished through the genetic modification of parent cells with GDNF-encoding plasmid DNA (*p*DNA). We characterized the obtained EV-GDNF by ELISA for the levels of GDNF and RT-PCR for genetic content, as well as by nanoparticle tracking analysis (NTA), atomic force microscopy (AFM) and Transmission Electron microscopy (TEM) for size distribution, charge, concentration, and morphology. Furthermore, the presence of specific proteins constitutively expressed in EVs was assessed by Western blot and label-free targeted quantitative proteomics. Next, we demonstrated that the intranasal (*i.n*.) administration route provided high accumulation levels of these nanocarriers in the brain [19]. Therefore, we used this route for the EV-GDNF treatment of Parkin Q311(X)A mice.

The novelty of this study can be outlined in three main points. First, we demonstrated that EVs can be loaded with GDNF protein and GDNF-encoding DNA through the genetic modification of EV-secreting macrophages. Second, label-free targeted quantitative proteomic analysis was applied for the first time to examine the effect of the genetic modification of parent cells on the expression and quantification of different integrins that play crucial roles in the accumulation of EVs in target cells. Third, prolonged, sustained therapeutic effects followed by *i.n.* administrations of EV-GDNF were demonstrated in PD mice over a year. Importantly, multiple lines of evidence for the therapeutic efficacy of EV-based formulations were observed, including decreased brain inflammation, significant neuroprotection of dopaminergic neurons in the SNpc brain region, and improved locomotor function. Finally, no overall toxicity was detected following administration of macrophage derived EVs.

## 2. Materials and Methods

### 2.1. Plasmids and Reagents

Human GDNF cDNA (NM_199234) was provided by OriGene (Rockville, MD, USA), which was propagated in DH5α E.coli, followed by purification using Giga-prep kits (Qiagen, Valencia, CA, USA). The sequence of hGDNF cDNA can be found on the web page: https://www.origene.com/catalog/cdna-clones/expression-plasmids/sc307906/gdnf-nm199234-human-untagged-clone (accessed on 15 November 2021) and the plasmid map for GDNF production is presented in Supplementary Figure S1. The structure of obtained plasmid was confirmed by electrophoresis with a single cut (AlwNI) and a double cut (AlwNI & PflMI) [38]. Murine macrophage-colony-stimulating factor (MCSF) was purchased from Peprotech Inc. (Rocky Hill, NJ, USA). Purified water was obtained from a Picopure^®^ 2 system (Hydro Service and Supplies, Inc., Durham, NC, USA). Trypsin Gold mass spectrometry grade (item # V5280) was purchased from Promega (Madison, WI, USA). Cell culture medium and fetal bovine serum (FBS) were purchased from Gibco Life Technologies, (Grand Island, NY, USA). Solid phase extraction (SPE) cartridges for sample clean-up (Strata™-X 33u Polymeric Reversed Phase, 10 mg/mL, part no. 8BS100AAK) were purchased from Phenomenex (Torrance, CA, USA). Fluorescent dyes, 1,1′-Dioctadecyl-3,3,3′,3′-Tetramethylindodicarbocyanine, 4-Chlorobenzenesulfonate Salt (DID), and 4′,6-diamidino-2-phenylindole (DAPI) were purchased from Invitrogen (Carlsbad, CA, USA). Ammonium bicarbonate; dithiothreitol; β-casein (from bovine milk); sodium deoxycholate; iodoacetamide: and acetic, formic, and trifluoroacetic acids were purchased from Millipore Sigma (St. Louis, MO, USA). Acetonitrile (HPLC grade), methanol and 0.2 mL flat-cap PCR tubes (catalog # 14230227) were purchased from Fisher Scientific (Pittsburg, PA, USA). All other chemicals were reagent grade.

### 2.2. Cells

To obtain autologous parental cells for the manufacture of GDNF-carrying EVs, female, wild-type littermates of the two-month-old transgenic mice were used as donors for bone-marrow-derived cells that were cultured in 75T flasks over 10 days in the presence of macrophage-colony-stimulating factor (MCSF, 1000 U/mL, at 37 °C and 5% CO_2_) [39]. The cells that did not attach to the flask during the culturing were considered non-differentiated cells and removed, with the media then replaced. The produced primary bone-marrow-macrophages (BMM) were characterized by flow cytometry with FACSCalibur (BD Biosciences, San Jose, CA, USA). The obtained data indicated that about 95% of the cells were in fact CD11b+.

### 2.3. Transfection Macrophages

Macrophages were transfected by electroporation. Briefly, 5 × 10^7^ cells were spun down at 125 RCF for 5 min, and then re-suspended in 700 µL electroporation buffer (Neon Transfection system, Thermo Fisher Scientific, Waltham, MA, USA) and supplemented with 30 µg GDNF-*p*DNA. The aliquot of the cell suspension with GDNF-*p*DNA (100 µL) was placed into an electroporation cell, and electroporated at the electroporation conditions outlined in Supplementary Table S1. Then, the cells were supplemented with 500 µL antibiotic-free media and cultured for up to 6 days in RPMI 1640 media (Sigma-Aldrich, St. Louis, MO, USA). At each time point, the EVs were isolated from the conditioned media, and the GDNF levels in the cells and EVs media were assessed by ELISA, as described earlier [40]. Sham macrophages were transfected with a sham vector: green fluorescence protein (GFP)-encoding *p*DNA.

### 2.4. EV Isolation

Concomitant media from GDNF-transfected macrophages was collected, and EVs were isolated using differential centrifugation [41]. First, the cells were purified from cell debris by sequential centrifugation at 300× *g* (10 min), then 1000× *g* (20 min), and finally 10,000× *g* (30 min), and subsequently filtered through 0.2 μm syringes. Next, the EVs pellet was obtained by centrifugation at 100,000× *g* (4 h), and washed with phosphate buffer solution (PBS). Of note, FBS was depleted from the FBS-derived EVs by centrifugation at 100,000× *g* (4 h) prior to the addition to the parent cells. The absence of EVs in FBS was confirmed by Nanoparticle Tracking Analysis (NTA). Bradford assay and NTA were used to estimate amount of recovered EVs released by GDNF-transfected macrophages [20]. To confirm that GDNF was incorporated/associated with EVs, and not co-simply co-precipitated with them, the obtained GDNF-EVs were purified by floating gradient centrifugation and GDNF levels in different fractions were assessed by ELISA as described earlier [41].

### 2.5. Characterization of EVs by Nanoparticle Tracking Analysis (NTA), and Atomic Force Microscopy (AFM), and Transmission Electron Microscopy (TEM)

EVs were collected from GDNF-transfected macrophage media, and the size and number of particles were evaluated using NanoSight 500, Version 2.2 (Wiltshire, UK). In addition, the concentration, and zeta potential (ZP) of the EVs were measured using a ZetaView QUATT Nanoparticle Tracking Microscope PMX-420 (Particle Metrix, Inning am Ammersee, Germany). For this purpose, the EVs were diluted to a concentration of about 2 × 10^7^ particles/mL, with 20 nm of filtered PBS. Measurements were performed at 11 positions using the following settings: maximum area 1000, minimum area 5, minimum brightness 20, camera level 16, and threshold level 5. Total protein was calculated using standard BCA assay. The AFM imaging was performed as described earlier [42]. A drop of isolated EVs in 50 mM phosphate buffer, pH 7.4, at total protein 10 µg/mL was placed on a glass slide and dried under an argon flow. For TEM imaging, EVs were adsorbed onto Formvar coated copper grid (400 mesh), stained with 2% uranyl acetate for 1 min. The samples were observed using a JEOL JEM-1230 transmission electron microscope operating at 80 kV (JEOL USA INC., Peabody, MA, USA) and images were taken using a Gatan Orius SC1000 CCD camera with Gatan Microscopy Suite version 3.10.1002.0 software (Gatan, Inc., Pleasanton, CA, USA).

### 2.6. Characterization of EV-Specific Proteins by Western Blot and Label-Free Targeted Quantitative Proteomics

The levels of proteins constitutively expressed in the EVs (CD9, CD63, CD81, TSG101, and HSP90) were identified by Western blot analysis, using Wes™ (ProteinSimple, San Jose, CA, USA). The EVs were lysed with 1× RIPA buffer for 30 min at room temperature, and 200 or 40 mg/mL of protein was denatured and loaded into Wes™ multi-well plates following the manufacturer’s instructions. Protein concentrations were determined using a BCA kit (Pierce Biotechnology, Rockford, IL, USA). For the analysis of the CD9, CD63, and CD81 samples, lysates were de-glycosylated using PNGase F PRIME (Bulldog Bio, Milledgeville, GA, USA, NZPP050) under non-denaturing conditions at a ratio of 1:9 v:v for 1 h prior to denaturalization. The protein bands were detected with the primary antibodies described in Supplementary Table S2 and secondary goat anti-rabbit HRP Conjugate (ready-to-use reagent, ProteinSimple, San Jose, CA, USA). Quantitative analysis of the obtained images was carried out using Compass SW software.

To characterize EV-GDNF by label-free targeted quantitative proteomics, the samples of EVs released by sham-transfected and GDNF-transfected macrophages were digested with trypsin, cleaned up by SPE, and prepared for nanoLC–MS/MS analysis, as described previously [43,44] with minor modification. For digestion, to 20 µg of solvent-evaporated EV protein was added 50 mM ammonium bicarbonate (100 µL), 40 mM dithiothreitol (10 µL), 0.5 mg/mL β-casein solution (10 µL) (as an indicator of successful digestion and to aid with chromatography retention time verification), and 13.3 µL of 10% sodium deoxycholate (to help with solubilization and denaturation). Samples were denatured for 40 min at 60 °C by shaking at 500 rpm in an Isotemp Thermal Mixer (Fisher Scientific). After cooling, 10 µL of 135 mM iodoacetamide was added, and the samples were incubated in the dark at room temperature for 30 min. Ten microliters (10 µL) of a solution containing 1 pmol of a Na^+^/K^+^-ATPase (membrane marker) stable-isotope-labeled (SIL) peptide standard (purchased from JPT Peptide Technologies, Berlin, Germany) in ~20/80 acetonitrile/50 mM ammonium bicarbonate was added to each sample. Trypsin (10 µL of 0.1 µg/µL solution in 50 mM acetic acid) was then added to give a 1/20 (*w*/*w*) trypsin/protein ratio. Samples were vortexed and digested at 37 °C for 20 h and shaken at 300 rpm in the Isotemp Thermal Mixer. After digestion, 10% TFA solution was added to stop the reaction, such that the volume added was 10% of the total volume of the digestion reaction. A deoxycholate precipitate formed. The precipitate was pelleted by centrifuging at 13,400× *g* (5 min). The obtained samples were purified on SPE cartridges with polymeric-reversed phase, 10 mg/mL, and eluted into LoBind Eppendorf tubes with 60% acetonitrile/40% formic acid. Then, the evaporated solution was reconstituted with 2% acetonitrile. The reconstituted sample was vortexed and then centrifuged at 13,400× *g* for 5 min. The supernatant was transferred to deactivated vial inserts (part # WAT094171DV; Waters, Milford, MA, USA) before nanoLC–MS/MS analysis.

The nanoLC–MS/MS analysis was performed on a nanoAcquity UPLC^®^ (Waters) coupled to a SCIEX QTRAP 5500 (Framingham, MA, USA) hybrid mass spectrometer with a NanoSpray^®^ III source. The specifics of this analysis have been previously reported in [23,43].

The sequences of peptides that could be identified and used in the final quantitative assessment are shown in Supplementary Table S3. UniProt accession numbers and the MRMs employed for each peptide have been previously published [23]. The test samples were analyzed in duplicate in the same batch, the method being both label- and standard-free.

### 2.7. Characterization of EV-GDNF by Quantitative qPCR Analysis

EVs or cells were lysed and directly added to qPCR reactions. qPCR was performed on lysates with 0.5 μL each of 20 μM forward and reverse primers 0.5–2 μL of the sample and 6.25 μL PowerUp SYBR Green Master Mix (Applied Biosystems, Waltham, MA, USA), with a total reaction volume of 12.5 μL, using a QuantStudio 6 Flex Real-Time PCR System (Applied Biosystems). Gene specific primers were used to amplify GDNF (forward sequence 5-GCAGACCCATCGCCTTTGAT-3 and reverse 5-CCACACCTTTTAGCGGAATGC-3).

### 2.8. Animals

Parkin Q311X(A) mice (2 breeding pairs, 12 weeks old) were obtained from Jackson Laboratory (Bar Harbor, ME, USA) and used to start a colony. The animals were treated in accordance with the Principles of Animal Care outlined by the National Institutes of Health and approved by the Institutional Animal Care and Use Committee of the University of North Carolina at Chapel Hill; “Inflammatory cells for transport of therapeutic polypeptides across the Blood Brain Barrier” ID# 21-030.0, Web ID: 90809, date for renewal 10 June 2022. PCR analysis was used for identification of transgenic mice and wild-type mice as described earlier [38]. All experiments were performed in 4–16-month-old male Parkin Q311X(A) mice on a C57BL/6 genetic background with age-matched, non-transgenic littermates serving as controls. Animals were housed in a temperature- and humidity-controlled facility on a 12 h light/dark cycle, and food and water were provided ad libitum. For all experiments, mice were monitored for any adverse signs of discomfort.

### 2.9. Treatment of Animals

Four groups of mice (*n* = 10) were employed in these studies. Three groups of Parkin Q311(X)A mice (4 mo. old) were intranasally injected with EV-GDNF (once a week, three weeks, 3 × 10^9^ particles/10 uL/mouse), or sham EVs (the same number of particles), or buffered saline (0.9% sodium chloride, pH = 7.4, negative control). Another group with wild type (WT) mice was injected with saline and used as a positive control. 

For intranasal injections (*i.n.*), each mouse was anesthetized with isoflurane (2% during induction, 1.5–2% during maintenance), until it showed no signs of reaction to a 4-paw toe pinch. Ample sterile ophthalmic gels were applied to ensure the moist state of the corneas of the animal. Then, the mouse was placed on a clean drape, facing up, with a heating pad underneath to maintain its body temperature. A padded pillow made of rolled-up paper towels with tape was adjusted to ensure the upright angle of the nostrils when it was placed under the head of the mouse. Using a micropipette, 5 μL of a treatment solution formulation was dispensed into each nostril of the mouse. The aspiration of the droplet was visually confirmed before moving to the next mouse. Animals were allowed to regain mobility in a recovery chamber, with the supine position maintained throughout. Mice were observed for recovery from sedation for 30 min after the *i.n.* drug administration. In particular, breathing, movement, and overall being were monitored.

### 2.10. Behavioral Studies

All 4 animal groups were subjected to standard behavioral tests before the treatment and for 1 year following the administration. To evaluate the therapeutic effects of EV-GDNF on locomotor activity and rearing movements, a wire-hanging task for grip strength, an accelerating rotarod procedure, and an open field test (OFT) were performed. Wire-hanging test for grip strength. Each mouse was placed on a wire, and the latency for the mouse to fall from the wire was recorded. The maximum trial length was 180 s. Rotarod test. For the traditional, constant-speed rotarod test, mice were trained and tested, as previously described with slight modifications [45]. The accelerating rotarod (Ugo Basil) was used for assessing motor coordination, balance, and ataxia. A rotarod machine with automatic timers and falling sensors was used. The mouse was placed on a 9 cm diameter drum. The surface of the drum was covered with hard chloroethylene, which does not permit gripping on the surface. Before the training sessions, the mice were habituated to stay on the stationary drum for 3 min. Habituation was repeated every day for 1 min just before the session. Mice were placed on a cylinder which slowly accelerates to a constant rotating speed. Normal mice readily learn to walk forward as the drum turns. For each trial, the revolutions per minute (rpm) are set at an initial value of 5, with a progressive increase to a maximum of 30 rpm across 5 min, the maximum trial length. For the first session, mice were given 3 trials, with 45 s between each trial. Measures were taken of latency to fall from the top of the rotating barrel. A second test session with 2 trials was conducted 48 h later to evaluate consolidation of motor learning. Activity in an open field. The hyperactivity of PD mice treated with different EV-based formulations, as well as WT mice injected with saline as controls (*n* = 10), was assessed by 5-minute and 1-hour trials in an open field chamber (41 cm × 41 cm × 30 cm), crossed by a grid of photobeams. Measures were taken by an observer blind to mouse genotype and treatment. Counts were taken of the number of photobeams broken during the trial, with separate measures for vertical rearing movements (VersaMax, AccuScan Instruments, Columbus, OH, USA). Time spent in the center regions was used as an index of anxiety-like behavior. Behavioral data were analyzed using one-way or repeated measures analysis of variance (ANOVA). For all comparisons, significance was set at *p* < 0.05.

### 2.11. Immunohistochemical and Stereological Analyses

At the endpoint, the 16-month-old animals were sacrificed and perfused. Postmortem brains were harvested, washed, and postfixed, and immunohistochemical analysis was performed in 30 µm-thick, consecutive, coronal brain sections. Two methods of sectioning the tissues were utilized. For investigations of the effect of EV-formulations on the protection of dopaminergic neurons, as well as decreases in microglial activation, the free-floating sections of the tissues were used. This approach allows the viewing of the structure of the dendrites and axons throughout the sample. The sections were not mounted on slides until after the completion of the immunohistochemistry process. To examine the neuroprotective effects of EV-based formulations, dopaminergic neurons were visualized with tyrosine hydroxylase staining as described in [46]. To study the microglial activation in the brains of the treated animals, primary monoclonal rat anti-mouse Mac1 antibodies (AbD Serotec, Raleigh, NC, USA) were used, along with secondary biotinylated goat anti-rat antibodies (Vector Laboratories, Burlingame, CA, USA). Each midbrain section was viewed at low power (10× objective), and the SNpc was outlined [47].

For the visualization of neurons, brain sections were stained with cresyl violet acetate solution (Nissl staining). For this purpose, mice were sacrificed and perfused, and the collected brains were frozen in optimal cutting temperature media (OCT). Frozen brains were cut into coronal sections with a 10 µm thickness using a CryoStarTM NX50 Cryostat (ThermoFisher, Waltham, MA, USA) and then placed on a warm pad to remove excess OCT. Slides were stored at −20 °C. A subset of sliced tissues was stained with cresyl violet acetate solution (Nissl) for the detection of Nissl bodies in the cytoplasm of neurons that stained purple-blue. In brief, tissues were exposed to xylene and rehydrated in a graded series of ethanol at 100, 95, and 75% concentrations. Sections were exposed to Nissl staining solution for 15 min, washed in distilled water, immersed in ethanol at 75%, 95%, and 100% concentrations, and then cleared by xylene. Slides were then mounted using mounting media for visualization. From 3 to 5 areas were randomly selected to be examined with an inverted fluorescence microscope and 63x objectives (Zeiss, Germany) by investigators who were blinded to the experimental groups. Another subset of sliced tissues was stained with hematoxylin and eosin (H&E) for the detection of morphological changes within the different brain regions. Tissues were exposed to xylene and rehydrated with ethanol at 100, 95, and 70% concentrations, followed by staining with hematoxylin dye for 15 min. After washes in distilled water, tissue sections were stained with eosin for 20 s and then dehydrated with gradient ethanol. Sections were exposed to xylene and then mounted using mounting media for visualization. Sections were imaged as described above.

### 2.12. ELISA

To examine the potential anti-inflammatory effects of EV-GDNF formulations in mice, the levels of the cytokines interferon γ (IFN-γ), interleukin 4 (IL-4), interleukin 6 (IL-6), interferon-inducible protein 10 (IP-10), and tumor necrosis factor alpha (TNF-α), and the chemokines monocyte chemotactic protein-1 (MCP1), regulated on activation, normal T-cell-expressed and secreted (RANTES), were analyzed in the spleen, brain, and liver recovered at necropsy. For this purpose, organs were removed post-mortem and homogenized in cell lysis buffer using an electrical homogenizer apparatus. Supernatant was used to measure the secretion levels of the inflammatory molecules by ELISA (R&D Systems, Minneapolis, MN, USA) according to the manufacturer’s instructions. The optical density was read at A450 on a Synergy HTX plate reader. Results are shown as the mean ± the standard error of the mean (SEM).

### 2.13. Statistical Analysis

For all experiments, data are presented as the mean ± SEM. Tests for significant differences between the groups in experiments regarding the characterization of EVs released by different types of parental cells were performed using a one-way ANOVA with multiple comparisons (Fisher’s pairwise comparisons) using GraphPad Prism, version 5.0 or higher (GraphPad software, San Diego, CA, USA). For targeted quantitative proteomics *data*, the MRM peak area data was processed with MultiQuant 2.0.2 software (SCIEX). Peak areas for the three highest-responding MRMs for each peptide were summed, and responses between samples were compared for each peptide. The presence of multiple peptides for a protein provided extra confidence that the protein/tetraspanin/integrin was present. SIL or label-free standards would be needed to compare the abundance of proteins within a sample, with large differences in signal sometimes being seen between peptides of the same amount. T-tests were used to determine whether peptide abundance differences between samples were significant (*p* < 0.05). For in vivo experiments, 10 mice were used per group. Thus, for behavioral tests, the total number of mice was determined by power analysis for the behavioral studies tests of the therapeutic effect of EV-mediated GDNF delivery from previously obtained by us data, wherein a = 0.05, s = 1640, and power = 0.90. To allow for equal sample sizes/group for ANOVA analysis, the number of mice necessary was 10 recipient mice/treatment group. This related to the variability of the inflammatory responses in mice. Next, for a histological evaluation of tyrosine hydroxylase positive neurons, numbers of recipients were determined using retrospective power analysis using the SAS JMP program. For dopaminergic loss, a 30% loss and subsequent neuroprotection from dopaminergic loss for =0.05, and =2251 with a power of 0.80 required 10 recipient mice/treatment group. For inflammatory responses, for example, a 30% diminution of inflammatory response by number of Mac-1+ reactive microglia/section for = 0.05, and = 4.6 with a power of 0.80 required 10 recipient mice/treatment group. For the evaluation of pro-inflammatory cytokines levels in PD mice, results are shown as the mean ± the standard error of the mean (SEM). For multiple factors, a two-way ANOVA followed by post-hoc tests as appropriate (Tukey’s or Dunnett’s) were used for multiple comparisons using Prism 8.0 (GraphPad Software). *p* < 0.05 was considered statistically significant.

## 3. Results

### 3.1. Manufacture of EV-GDNF by Genetic Modification of Primary Macrophages

In this work, we utilized the endogenous loading of EVs through the parent cells. Based on our previous investigations [23,27,47,48,49], primary macrophages were selected for the production of the EV-GDNF formulation. Briefly, macrophages were transfected with GDNF-encoding plasmid DNA (*p*DNA) by electroporation at different electroporation conditions (as described in the Section 2), and the levels of GDNF in parent cells and in EVs collected from macrophage-conditioned media were assessed by ELISA for 6 days after the transfection (Supplementary Figure S2). Significant levels of GDNF were detected in the transfected macrophages (solid bars), as well as EVs isolated from the media (striped bars). Of note, the GDNF expression levels in the macrophages gradually decreased from day 1 to day 6 although the amount of GDNF in EVs increased, especially in later days. The variation of electroporation conditions (#2–#4) did not significantly affect the GDNF amount in the EVs, so condition #4, which provided slightly higher GDNF content in the EVs, was selected for further investigations.

To confirm that GDNF was incorporated/associated with EVs and not simply co-precipitated with them, the GDNF-EVs were purified by floating gradient (Supplementary Figure S3A) and EVs and non-EVs containing fractions were examined by ELISA (Supplementary Figure S3B). The obtained results confirmed that fraction containing EVs showed considerable amount of GDNF. Of note, there was some GDNF isolated from the fraction that does not contain EVs. However, the amount of free GDNF was significantly lower than those associated with EVs. We speculated that free GDNF might be released from GDNF- transfected parent cells or from GDNF-EVs. No GDNF was found in control EVs fraction released by sha-transfected macrophages. The purified EV-GDNF were utilized in all further experiments.

### 3.2. Characterization of EV-GDNF

**Figure 1:**
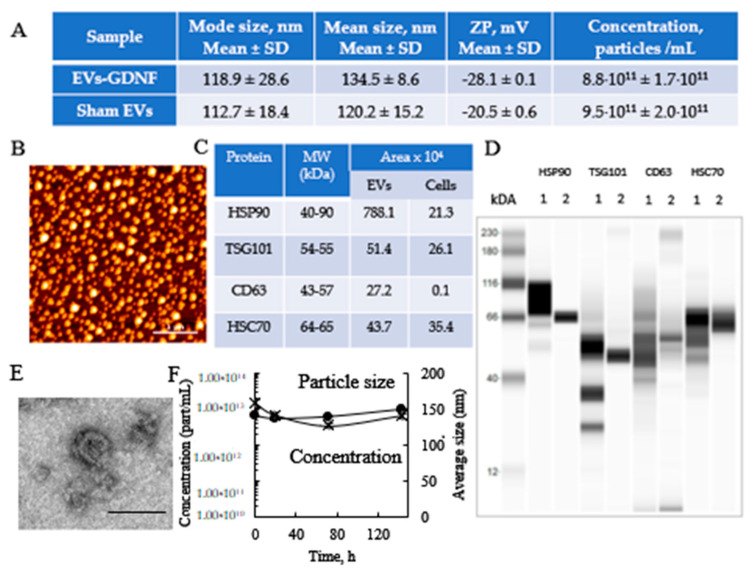
Characterization of EV-GDNF by ZetaView QUATT Nanoparticle Tracking Microscope PMX-420, AFM, TEM, and Western blot. Primary macrophages were transfected with GDNF-encoding pDNA by electroporation (condition #4), and EV-GDNF were collected from conditioned media on day 6. EV-GDNF were characterized for size, zeta potential, morphology, and stability by ZetaView QUATT Nanoparticle Tracking Microscope PMX-420 (**A**), AFM (**B**), and TEM (**E**). The abundance of EV-specific membrane proteins in GDNF-EVs (**1**) and much less—in parent macrophages (**2**) was confirmed by Wes^TM^ (**D**) and quantified using Compass SW software (**C**). High stability of EV-GDNF nanoparticles was demonstrated over a week by NTA (**F**). The bar: 1 µm for AFM (**B**) and 100 nm for TEM (**E**).

EV-GDNF collected from genetically modified parent macrophages were characterized for size, charge, shape, and morphology. Sham EVs collected from sham transfected macrophages were used as a control. As seen on Figure 1, the transfection of parent cells with GDNF-encoding *p*DNA did not significantly affect size and charge of the nanocarriers. According to NTA data, average mode size 120 nm with negative ZP around -20 mV was recorded for both EV-GDNF and sham EVs (Figure 1A). Next, the spherical morphology of the obtained EV-GDNF with relatively uniformly size distribution was confirmed by AFM (Figure 1B). More detailed structure was obtained by TEM (Figure 1E). Finally, the presence of EV-specific proteins (SHP90, TSG101, CD63, and SHP70) in EV-GDNF was confirmed by Wes™ Simple Western Blot (Figure 1D) and quantified using Compass SW software (Figure 1C), confirming that these proteins were enriched in EVs fraction (**1**) compared to the cell lyzate (**2**). High stability of EV-GDNF nanoparticles was demonstrated over a week by NTA (Figure 1F).

We reported earlier that, similar to their parent cells, macrophage derived EVs exert unique biological activity reflective of their origin that allows them to easily penetrate the BBB and migrate rapidly to sites of neurodegeneration [50]. It is of paramount importance in the view that chronic brain inflammation is a common feature for different neurodegenerative disorders, including PD patients [51]. In addition, EVs have different adhesive proteins expressed on their membranes that allow efficient binding and delivery of their cargo to target cells [52]. However, genetic modification of parent macrophages may alter their composition. Therefore, we investigated the expression of the EV-specific proteins in the EV-GDNF by label-free targeted quantitative proteomics. Specifically, we assessed the relative expression levels of adhesive tetraspanins (CD63 and CD9), ALIX, integrin β-1 (CD29), and tsg101 released by GDNF-transfected macrophages and compared with those released by sham-transfected cell (See Supplementary Table S3). Additionally, we studied the presence of α integrins and integrin β-2 that facilitate targeting to the inflamed endothelium. The data revealed that the transfection of parent macrophages did not statistically significantly affect the expression of the proteins of interest (Supplementary Figure S4), suggesting that, similar to sham EVs, EV-GDNF could successfully deliver therapeutic cargo to the inflamed brain.

**Figure 2:**
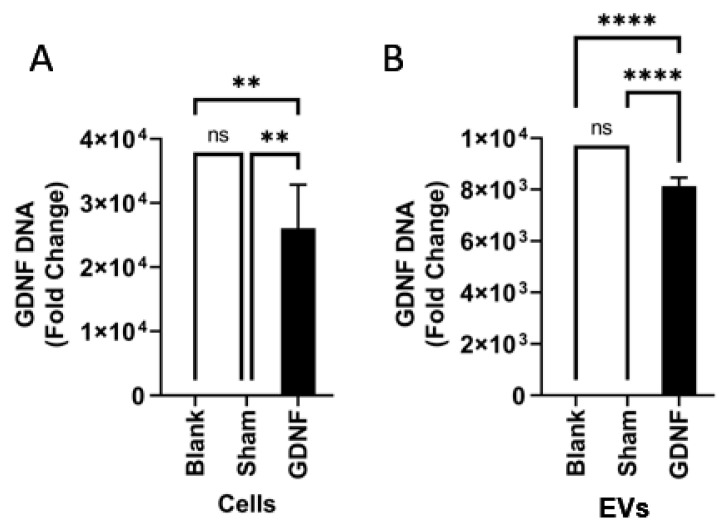
Characterization of genetic content of GDNF-transfected parent macrophages and released EV-GDNF by quantitative qPCR. Macrophages were transfected with GDNF-encoding *p*DNA by electroporation, and the levels of GDNF-DNA in the cells (**A**) and EVs released by these cells (**B**) were assessed. A significant amount of GDNF-DNA was detected in parent cells, as well as in the EVs. Statistical significance was assessed by one-way ANOVA corrected for multiple comparisons using the FDR. ** *p* < 0.01, or **** *p* < 0.0001.

Finally, the presence of GDNF-encoding DNA in EVs released by GDNF-transfected parent cells was studied by qPCR analysis (Figure 2). The obtained data suggested that these EVs contained a significant amount of GDNF-DNA. This result is consistent with our previous reports [21,53], indicating that EVs secreted from GFP- and TPP1-transfected parent macrophages contain corresponding genetic material.

### 3.3. Effect of EV-GDNF on Locomotor Activity upon Intranasal Administration to Parkin Q311(X)A Mice

**Figure 3:**
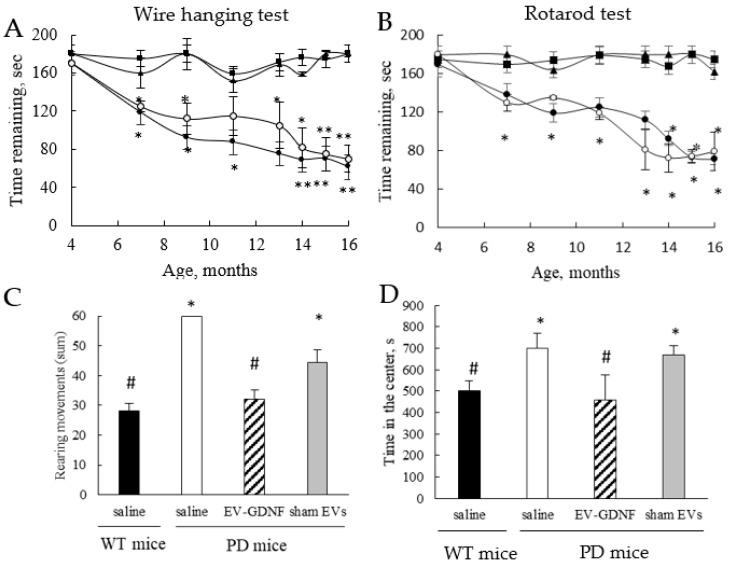
Behavioral tests demonstrating significant therapeutic effect of EV-GDNF in Parkin-Q311X(A) mice. The effect of EV-GDNF on motor functions and activity was assessed in a wire-hanging test and rotarod test (**A**,**B**), as well as in OFA tests (**C**,**D**). (**A**,**B**) Transgenic mice were *i.n*. injected with EV-GDNF (triangles, 3 × 10^9^ particles/10 µL/mouse), or sham EVs (empty circles, 3 × 10^9^ particles/10 µL/mouse), or saline (filled circles, 10 µL/mouse). Wild-type mice *i.n*. injected with saline (filled squares, 10 µL/mouse) were used as controls. Wire-hanging test (**A**), and rotarod test (**B**) demonstrated significant improvements in motor functions upon treatment with EV-GDNF. (**C**,**D**) OFA tests at 12 mo demonstrated improved behavior in EV-GDNF-treated PD mice (striped bars) compared to PD mice treated with saline (white bars), which was similar to healthy WT mice (black bars), including decreases in hyperactivity and anxiety-like behaviors. The differences between sham EVs and saline in PD mice were inconclusive. Values are means ± SEM (*n* = 10), * *p* < 0.05, ** *p* < 0.005, and # *p*< 0.05, as compared to WT control mice injected with saline.

Based on our previous reports [19], we selected the intranasal (*i.n*.) administration route for treatment with EV-based formulations as one of the most efficient routes for the brain delivery. Transgenic PD mice (4 mo old) were treated with EV-GDNF (3 × 10^9^ particles/10 µL/mouse) once a week, 3 times, and their locomotor functions were assessed in a battery of behavioral tests (Figure 3). PD mice and wild-type (WT) counterparts treated with saline were used as positive and negative controls, respectively. PD mice injected with sham EVs were used in another control group. The behavioral tests, including the wire-hanging test (Figure 3A) and rotarod test (Figure 3B), were performed for all animal groups over 1 year.

The obtained data indicated the significant therapeutic efficacy of EV-GDNF treatments reflected in preservation of locomotor functions in PD mice (Figure 3A,B; triangles) compared to PD mice treated with saline (Figure 3A,B; filled circles). Moreover, the remaining time in the wire-hanging test and the latency to fall in the rotarod test were almost the same as in the WT healthy animal group (Figure 3A,B; squares). Of note, the preservation of locomotor activity in PD mice by EV-GDNF was recorded for as long as 1 year, and it was almost the same as in healthy WT animals (Figure 3A,B; filled squares). No statistically significant effects were detected in the transgenic mice treated with sham EVs (Figure 3A,B; empty circles), indicating these comparisons were inconclusive.

To reinforce this conclusion, we performed open field activity (OFA) tests with 16-month-old animals (Figure 3C,D). A one-hour trial was completed in an OF chamber (41 cm × 41 cm × 30 cm) equipped with a crossing grid of photobeams (VersaMax system, AccuScan Instruments) to assess the effect of therapeutic treatments on the hyperactivity of PD mice. The number of rearing movements of the animals, and the amount of time spent in the center of the chamber, a known index of anxiety-like behavior, were recorded. The OFA tests indicated that PD mice treated with saline (white bars) showed hyperactivity with more errors per step while traversing the beam (Figure 3C), and anxiety-like behavior manifested in more time spent in the center region (Figure 3D) compared to WT mice treated with saline (black bars). In contrast, PD mice treated with EV-GDNF (striped bars) displayed improved behavior that was similar to healthy WT mice treated with saline. Whether the treatment with sham EVs (grey bars) affected the performance patterns of PD mice was inconclusive, and the PD mice demonstrated similar hyperactivity and anxiety-like behaviors as control PD animals treated with saline.

### 3.4. Neuroprotective and Anti-Inflammatory Effects in ParkinQ311(X)A Mice

**Figure 4:**
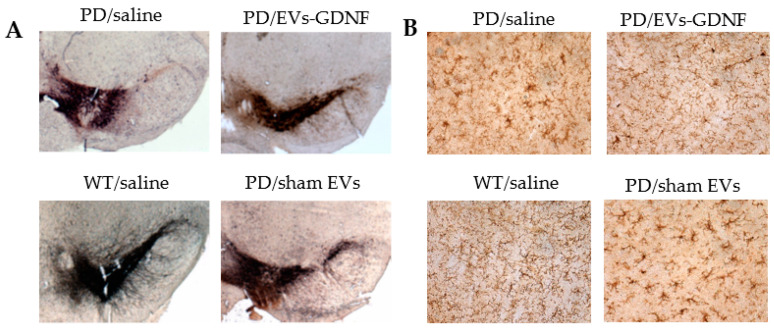
Neuroprotective and anti-inflammatory effects of EV-GDNF in Parkin Q311(X)A mice. Transgenic mice (4 mo old, *n* = 10) were *i.n.* injected with: saline (10 µL/mouse), EV-GDNF (3 × 10^9^ particles/10 µL/mouse), or sham EVs (3 × 10^9^ particles/10 µL/mouse). Wild-type control mice were intranasally injected with saline (10 µL/mouse). Animals were sacrificed at month 16, and brain slides were stained with TH, a marker for dopaminergic neurons (**A**); or Ab to CD11b for activated microglia (**B**). The images indicate significant preservation of TH-positive neurons and a decrease in microglial activation in Parkin Q311(X)A mice upon EV-GDNF treatment compared to PD mice treated with saline. The administration of sham EVs did not cause significant therapeutic effects.

A total of 1 year after the first treatment, the mice (16 mo old) were sacrificed, their brains were isolated, and the sections were mounted on slides. The mid-brain slides were stained for the expression of tyrosine hydroxylase (TH), a marker for dopaminergic (DA) neurons (Figure 4A, Supplementary Table S4). The almost-complete degeneration of DA neurons in the SNpc was observed in PD mice treated with saline compared to the healthy WT mice. In contrast, treatments with EV-GDNF dramatically ameliorated PD-related neurodegeneration. Furthermore, potent anti-inflammatory effects of EV-GDNF were demonstrated (Figure 4B, Supplementary Table S4). Thus, the neurodegeneration in 16-month-old Parkin Q311(X)A mice treated with saline was accompanied by substantial brain inflammation as displayed by the up-regulated expression of CD11b by microglia within the SNpc, which exhibited a more amoeboid morphology with larger cell body, as compared to the WT mice treated with saline. Importantly, the treatment with EV-GDNF significantly reduced neuroinflammation in PD mice according to decreased microgliosis revealed by ramified microglia. No anti-inflammatory effects of sham EVs were found in PD mice. Additional images of brain slides with staining to TH-positive neurons and activated microglia are presented on Supplementary Figures S5 and S6, respectively.

**Figure 5:**
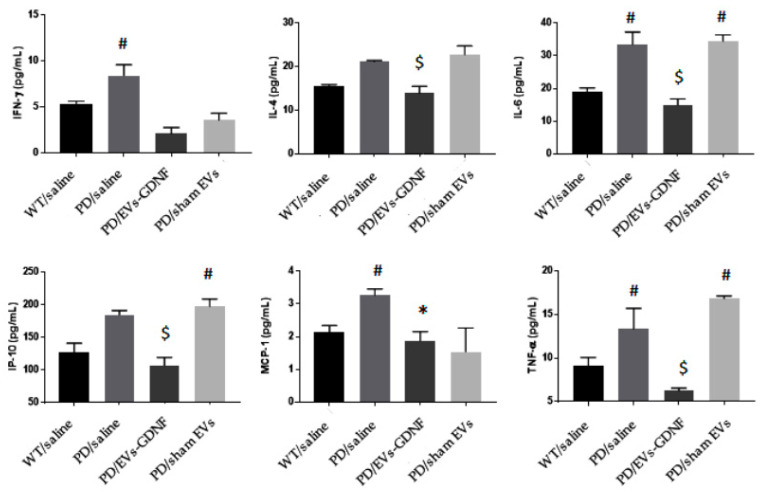
Anti-inflammatory effects of GDNF-carrying EVs in PD mouse model. Transgenic mice (4 mo old) were intranasally injected with: saline (10 µL/mouse), or EV-GDNF (3 × 10^9^ particles/10 µL/mouse), or sham EVs (3 × 10^9^ particles/10 µL/mouse). Wild-type control mice were intranasally injected with saline (10 µL/mouse). Animals were sacrificed at month 16, brains were removed post-mortem, and they were homogenized in cell lysis buffer. Elevated cytokine levels in the brain were recorded in PD mice treated with saline and sham EVs. Administration of EV-GDNF significantly decreased pro-inflammatory molecules in the brains compared with PD mice treated with saline. *n* = 4; # *p* < 0.05 compared to healthy WT animals; * *p* < 0.05 compared to PD mice treated with saline; ^$^ *p* < 0.05 compared to PD mice treated with saline and sham EVs.

Furthermore, the anti-inflammatory effects of the EV-GDNF treatments were confirmed by measuring the levels of pro-inflammatory cytokines and chemokines in main organs of the animals (Figure 5). Significant (#) secretion of IFN-γ, IL-6, MCP-1, and TNF-α were identified in the brains of PD mice injected with saline, and of IL-6, IP-10, and TNF-α in sham EVs, when compared to healthy controls. In contrast, PD mice treated with EV-GDNF showed a significant decrease (*) in secretion of IFN-γ and MCP-1 when compared to PD mice treated with saline, and a significant decrease ($) in IL-4, IL-6, IP-10, and TNF-α, when compared to PD mice treated with saline and sham EVs, similar to WT mice, or even lower. This confirms that EVs loaded with GDNF could diminish inflammation in the brains of PD mice.

**Figure 6:**
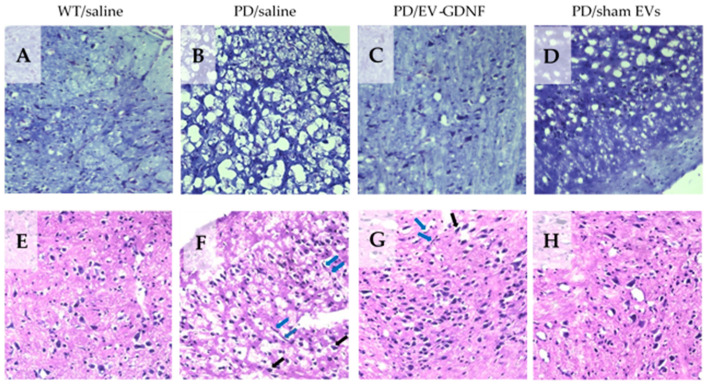
Histological analysis of neuroprotective effects of EV-GDNF in Parkin Q311(X)A mice. Transgenic mice (4 mo old) were intranasally injected with: saline (10 µL/mouse), (**3**) EV-GDNF (3 × 10^9^ particles/10 µL/mouse), or sham EVs (3 × 10^9^ particles/10 µL/mouse). Wild-type control mice were intranasally injected with saline (10 µL/mouse). Animals were sacrificed at month 16, and brain slides were stained with Nissl staining (**A**–**D**) or H&E staining (**E**–**H**). The obtained bright-light images show lower numbers of Nissl bodies with neuronal shrinkage (**B**) and damaged tissues with degeneration in the neurons (**F**) in PD mice treated with saline when compared to WT mice (**A**,**E**). Histological analysis indicates neuroprotective effects in the brain of PD mice treated with EV-GDNF with healthy morphology in tissue structure and high integrity of neurons (**C**,**G**) when comparted to PD mice treated with saline (**B**,**F**). The administration of sham EVs did not have a significant therapeutic effect in PD mice (**D**,**H**). Black arrows, degenerated neurons; blue arrows, elongated irregular nuclear morphology.

The neuroprotective effects of EV-based formulations in Parkin Q311(X)A mice were further confirmed with Nissl staining (Figure 6A–D). While a large quantity of healthy neurons was found in the SNpc of WT mice (Figure 6A), PD mice treated with saline showed lower numbers of neurons and astrocytes (Figure 6B). In contrast, brains of PD mice treated with EV-GDNF showed better tissue integrity, with minimal signs of vacuolation in neurons (Figure 6C). Of note, treatments with sham EVs did not have this therapeutic effect (Figure 6D). The same effect of neuronal protection in PD mice by EV-GDNF was shown by H&E staining (Figure 6E–H). In particular, healthy cell morphology and low vacuolation was detected in WT mice (Figure 6E), while damaged tissue with degeneration in the neurons (black arrows) and elongated irregular nuclear morphology of microglia (blue arrow) were observed in the brain slides of PD animals treated with saline (Figure 6F). Moreover, the vacuolation within the overlying molecular layer suggests potential swelling/degeneration of Purkinje neuron dendrites. Healthier-looking tissues with a high integrity of neurons visible in the tissues of mice treated with EV-GDNF (Figure 6G) indicated neuroprotection, compared to PD mice treated with saline (Figure 5G). However, elongated nuclear morphology of microglia (blue arrow) and some necrotic neurons (black arrows) were detected, albeit noticeably less than in PD animals treated with saline (Figure 6F). In accordance with all other assays, sham EVs did not produce significant therapeutic effects in PD mice (Figure 6D,H). Additional images of brain slides with Nissl staining and H&E staining are presented in Supplementary Figures S7 and S8, respectively.

Finally, the possible inflammatory effects of EV-GDNF formulations were also examined in the main peripheral organs, liver, and spleen. We demonstrated earlier that these organs accumulate the most amounts of EVs in mice [21,23]. For this purpose, levels of the pro-inflammatory cytokines IFN-γ, IP-10, IL-4, IL-6, RANTES, MCP-1, and TNF-α were assessed in PD mice treated with EV-GDNF and compared with those in WT animals treated with saline (Supplementary Table S5). No significant increases in cytokine levels were found following the administration of EV-GDNF. This is consistent with our previous reports, which indicated no significant toxicity of EVs [54]. Furthermore, no total weight loss was detected in mice treated with EV-GDNF or sham EVs compared to those treated with saline (Supplementary Figure S9). This signifies an absence of general toxicity of the cell-based formulations upon multiple administrations of EVs treatments.

## 4. Discussion

In this work, we exploited macrophage-derived EVs as promising bio-nanostructures that allow for the targeting of disease tissues and the efficient delivery of incorporated therapeutics to the brain. We posit that this approach has the potential to become a novel treatment strategy that links a cutting-edge class of precision medicines with the latest nanotechnology. We reported the earlier development of EV-based therapeutic formulations using the exogenous loading of naïve EVs with a number of potent therapeutic proteins, including the antioxidant enzyme catalase [19]; brain-derived neurotrophic factor (BDNF) [22]; and tripeptidyl peptidase-1 (TPP1), a therapeutic enzyme for the treatment of a lysosomal storage disorder, Batten disease [21]. However, in some cases, therapeutic proteins may be not available in significant amounts for exogenous loading into EVs. Furthermore, many therapeutic molecules, especially bioactive proteins and enzymes, are highly susceptible to degradation or deactivation upon loading. Herein, we utilized a different strategy, the endogenous loading of GDNF through the transfection of parent cells with GDNF-encoding *p*DNA.

First, we optimized transfection conditions, which allowed for the efficient genetic modification of parent autologous macrophages and manufactured a considerable amount of EVs with the prolonged expression of GDNF. We characterized the obtained EV-GDNF according to MISEV2018 guidelines for size, charge, shape, morphology, and protein content. Spherical nanoparticles, with a relatively uniform size around 120 nm, were confirmed by NTA analysis and AFM. The size of the particles appeared to be on the upper side of the typical median particle size to ensure successful deposition within the nasal cavities. It has been reported that particles larger than 130 nm may deposit at the front of the nose, while finer particles tend to penetrate further into the brain tissues [55]. Furthermore, the presence of EV-specific proteins that are known to facilitate EV adhesion to target cells was verified for the EV-GDNF by Western blot.

It is of paramount importance to ensure that GDNF was incorporated/associated with EVs in the developed formulation and not just co-isolated with these drug carriers. To this point, we reported the earlier characterization of EVs isolated from GDNF-transfected macrophage media by Western blot [40]. We demonstrated that GDNF was protected in EVs against degradation by pronase. At the same time, free GDNF was completely degraded under the same conditions. The destruction of EVs by sonication eliminated this protective effect. This indicates that at least a significant portion of the GDNF molecules was incorporated into the EVs. Nevertheless, we cannot completely state that all of the GDNF molecules were in the EV lumen; some portion could be associated with the EV membrane. Furthermore, we demonstrated here that, along with the encoded therapeutic protein (GDNF), EVs released by pre-transfected macrophages contain genetic material, GDNF-DNA. This may result in the transfection of brain tissues and GDNF expression at the disease site. We speculated that a specific mechanism that results in the DNA-targeted accumulation of EVs might exist in the parent cells. Further investigations regarding the specific location of GDNF on EVs and the possibility of transfection of brain tissues by EV-GDNF are ongoing in our lab.

Regarding the targeting of drug delivery by EV nanocarriers, it is well-established that the specialized cells of the immune system, including monocytes, macrophages, and T cells, can accumulate in the PD brain, migrating to the sites of neuroinflammation and degeneration [56,57]. Moreover, this ability to target inflamed tissues was also shown for macrophage-derived EVs [19,21,36], which is of particular importance due to the fact that the pathological process in the brain of PD patients is accompanied with chronic neuroinflammation [51]. Herein, using label-free targeted quantitative proteomics, we confirmed that genetic modification of parent macrophages with GDNF-encoding *p*DNA did not significantly alter the expression levels of specific integrins on EVs that promote adhesion and targeting tissues with inflammation. This suggests that the obtained EV-GDNF would accumulate in the PD mouse brain and potentially deliver their therapeutic cargo to the disease site.

For the assessment of the therapeutic efficacy of EV-GDNF in a transgenic mouse model of PD, we initiated treatments via intranasal (*i.n*.) administration at early stages of the disease. This route provides two different paths for transport to the brain. These are: (i) the passage along the olfactory nerve cells, where nanoparticles bypass the BBB and enter the brain directly; and (ii) the transport across the epithelial cell layer to the systemic blood circulation, and then transferring across the BBB into the brain parenchyma. Of note, the first route allows entrance to the CNS without first-pass hepatic and intestinal metabolism, which may significantly reduce EV clearance in these peripheral organs [58]. Thus, the mucosa and lamina propria are exceedingly vascularized with the high-absorption-rate epithelium [59]. Furthermore, this non-invasive route of drug delivery has high patient compliance and does not require frequent hospital visits. We also demonstrated earlier that a considerable amount of EVs administered through the *i.n.* route accumulated in the mouse brain with neuroinflammation [19]. Thus, confocal images of PD mouse brains showed diffuse staining of fluorescently labeled EVs, along with vesicular compartments localized predominantly in perinuclear regions 4 h after *i.n.* administration. Therefore, the nasal route is receiving considerable attention for administering drugs that net systemically. Nevertheless, it should be noted that there are several limitations, including the limited volume that can be dropped or sprayed into the nasal cavity, as well as the removal of the drug by mucociliary clearance. Interestingly, some investigators hypothesized that PD actually has its origin in the bulbs olfactory and then spreads thought the brain, ascending cell-by-cell through the brainstem, midbrain, and other regions of the brain [60]. Thus, we reasoned that *i.n.* administration of EV-GDNF may work in the same manner as natural disease spread, delivering therapeutic GDNF to the most affected brain areas.

Parkin Q311(X)A mice were treated at month 4, weekly, 3 times, and their locomotor functions were assessed over 1 year. Notably, the *i.n.* treatments with EV-GDNF significantly improved the mobility of PD animals, compared to PD groups treated with saline or sham EVs, up to the levels in healthy WT mice. Along with improved mobility, OFA studies showed substantial improvements in behavior patterns, including decreases in hyperactivity and anxiety-like behaviors. At the end-point, the mice were sacrificed, and the neuroprotection effects and decreases in oxidative stress in the brains of PD animals treated with EV-GDNF were confirmed by histological evaluations. Specifically, EV-GDNF treatments produced significant neuroprotection and reduced neuroinflammation in Parkin Q311(X)A mice. These findings could be of great importance for clinical applications of EV-based treatments. Regarding the possible mechanisms of action of EV-based formulations in PD mice, GDNF is known to interact with a receptor of GDNF family, GFRA2 [61]. We hypothesized that EV nanocarriers would protect GDNF against degradation and facilitate the delivery of this therapeutic protein to the brain tissues. Some of GDNF molecules may be incorporated into the EV lumen, and some of them—associated with the EV outside membranes. However, in order to produce its therapeutic effect, GDNF should be released from the EVs upon arrival at the target tissues and interact with GFRA2 receptor. Therefore, the absence of a strong attachment to the EV membrane, which allows GDNF molecules to be released in the brain, may be preferable. Of note the superior therapeutic effects of EV-GDNF in PC12 neurons, which are known to express the GDNF receptor, as compared to a high concentration of GDNF alone, were shown earlier in vitro studies by confocal microscopy [40]. Specifically, we reported a pronounced outgrowth of axons and dendrites in neurons cultured with EV-GDNF, which was greater than that caused by a high dose of commercially available GDNF.

To further boost the therapeutic effect of EV-based formulations and ensure an absence of side effects, we utilized anti-inflammatory M2-subtype of macrophages for manufacture EV-GDNF. We reported earlier [19] that EVs can reflect the properties of their parent cells and carry the same signal molecules. For example, we demonstrated that EVs released from M2 anti-inflammatory macrophages express the M2 subtype markers, Arg1 and CD206. In contrast, EVs released by M1 macrophages carry pro-inflammatory markers, such as iNOS. Of note, the genetic modification of M2-macrophages did not change their subtype [19], which was confirmed on brain slides of PD mice injected with pre-transfected cells. We also report here the lack of any systemic toxicity followed EV-GDNF treatments.

Notably, EV-GDNF interventions resulted in a prolonged abrogation of neurodegeneration and neuroinflammation by EV-GDNF treatments in PD animals. We speculated that these sustained therapeutic effects may be attributed to the fact that, along with GDNF, EVs released by GDNF-transfected macrophages carry genetic material encoding this therapeutic protein, GDNF-DNA. This may result in the transfection of brain tissues and the overexpression of GDNF, which would explain these prolonged therapeutic effects. Indeed, one of the natural functions of EVs is the transfer of genetic information to nearby and distant organs and tissues, including the transfer of both coding and non-coding RNAs to recipient cells [62]. We reported earlier that EVs released by pre-transfected parent cells contain an encoded therapeutic or reporter protein (i.e., catalase, TPP1, luciferase, or green fluorescence protein), along with the *p*DNA encoding these proteins. Moreover, we found in EVs a transcription factor that was involved in the encoded gene expression (i.e., NF-kb) [53]. Thus, endogenously loaded EV-GDNF may represent a potent, non-immunogenic, naturally manufactured kit for the transfection of disease tissues. We hypothesized that the accumulation of EV-GDNF in brain tissues may result in the transfer of the EVs’ cargo to the tissues and the de novo synthesis of the encoded protein in target cells. As such, EVs obtained from genetically modified parent cells represent a novel class of vectors that may accomplish gene transfer at the distant organs, similar to their natural functions. The mechanistic studies regarding this mechanism are ongoing in our lab and will be reported in future publications.

Collectively, our data indicates that EV-based formulations of GDNF are a promising therapeutic modalities that can provide versatile and potent strategies for the CNS delivery of therapeutics which could be applied to different neurodegenerative disorders. Overall, the exploration of mechanisms involved in the targeted CNS transport of EV-based drug formulations is crucial for therapeutic applications and transformation the field of precision medicine.

## Data Availability

Not applicable.

## Supplementary Materials

The following are available online at https://www.mdpi.com/article/10.3390/cells11121933/s1, Figure S1: Absence of gross toxicity of EV-GDNF treatment in Parkin Q311(X)A mice; Figure S2: Transfection of primary murine macrophages with GDNF encoding *p*DNA; Figure S3: GDNF levels in EVs released by genetically modified macrophages by ELISA; Figure S4: Quantification of Integrins and Tetraspanins in EVs by Label Free Targeted Quantitative Proteomics; Figure S5: Neuroprotective effects of EV-GDNF in Parkin Q311(X)A mice; Figure S6: Anti-inflammatory effects of EV-GDNF in Parkin Q311(X)A mice; Figure S7: Histological analysis of neuroprotective effects by EV-GDNF in Parkin Q311(X)A mice; Figure S8: Histological analysis of neuroprotective effects by EV-GDNF in Parkin Q311(X)A mice; Figure S9: Absence of gross toxicity of EV-GDNF treatment in Parkin Q311(X)A mice. Table S1: Operation parameters of parent macrophage electroporation upon transfection with GDNF-encoding *p*DNA; Table S2: Primary antibodies used for Simple Western blot; Table S3: Expression levels of EV-specific proteins in EVs released by GDNF-transfected macrophages; Table S4: Murine integrin proteotypic tryptic peptides detectable by label-free quantitative assessment; Table S5: Effect of EV-GDNF on inflammation and neurodegeneration in PD mice.
