# Supplementary material for: Using Extracellular Vesicles Released by GDNF-Transfected Macrophages for Therapy of Parkinson Disease
Source: Cells. 2022 Jun 15;11(12):1933. doi: 10.3390/cells11121933 (PMC9222008; doi:10.3390/cells11121933)
Supplement: Supplementary file 1 [file cells-11-01933-s001.zip › cells-1523698-supplementary.pdf]

**Supplementary Materials**

# Using Extracellular Vesicles Released by GDNF-Transfected Macrophages for Therapy of Parkinson Disease

**Yuling Zhao [1,2], Matthew J. Haney [1,2], John K. Fallon [2], Myosotys Rodriguez [3], Carson J. Swain [2], Camryn J. Arzt [2], Philip C. Smith [2], Matthew Shane Loop [2], Emily B. Harrison [2], Nazira El-Hage [3] and Elena V. Batrakova [1,2,*]**

Center for Nanotechnology in Drug Delivery, University of North Carolina at Chapel Hill, Chapel Hill,
NC 27599, USA; yulingz@email.unc.edu (Y.Z.); mjhaney@email.unc.edu (M.J.H.)

[2] Eshelman School of Pharmacy, University of North Carolina at Chapel Hill, Chapel Hill, NC 27599, USA; jfallon@email.unc.edu (J.K.F.); cswain@unc.edu (C.J.S); camiarzt@live.unc.edu (C.J.A.); pcs@email.unc.edu (P.C.S.); mloop@email.unc.edu (M.S.L.); emilybrookeharrison@gmail.com (E.B.H.)

[3] Herbert Wertheim College of Medicine, Florida International University, FL 33199; myrodrig@fiu.edu (M.R.); nelhage@fiu.edu (N.E.-H.)

*Correspondence should be addressed to E.V.B. (batrakov@ad.unc.edu)

UNC Eshelman School of Pharmacy, University of North Carolina at Chapel Hill, Chapel Hill, NC 27599-7362, Phone: 919-537-3712, Email: batrakov@ad.unc.edu

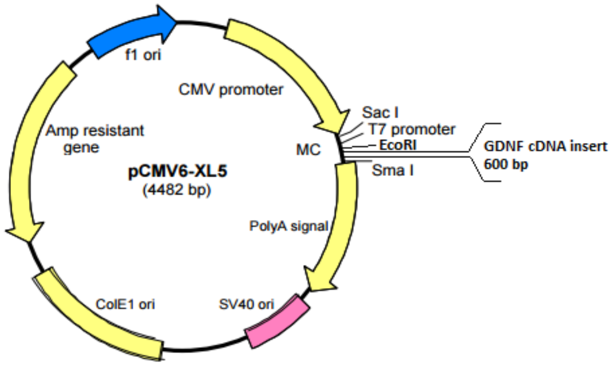


**Supplementary Figure S1. Plasmid map for GDNF production**

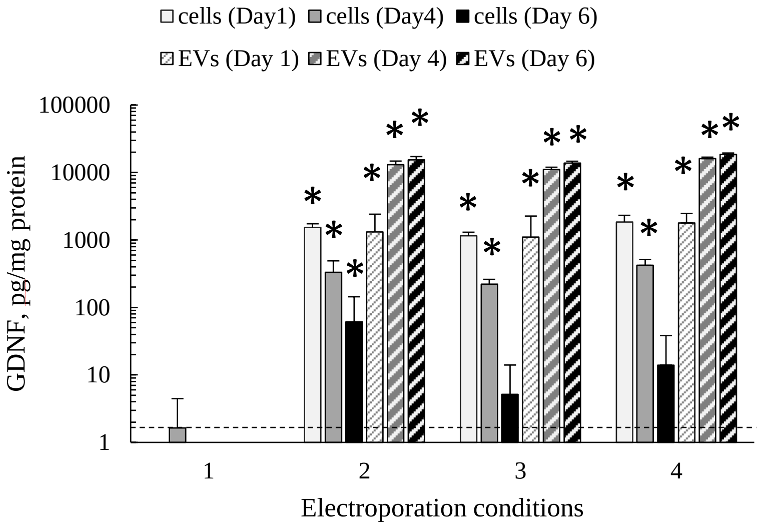


**Supplementary Figure S2. Transfection of primary murine macrophages with GDNF-encoding *p*DNA.** Bone-marrow derived macrophages were transfected by electroporation using four different conditions described in Experimental section. Then, cells were washed and cultured in complete media for up to 6 days. The GDNF expression levels in cells (solid bars), and EVs collected from conditioned media (stripped bars) was assessed by ELISA on day 1 (white bars), day 4 (grey bars), and day 6 (black bars). Successful transfection was accomplished with three conditions (#2 - #4). n = 4, *p < 0.05, compared to sham-transfected macrophages (dashed line, condition #1).

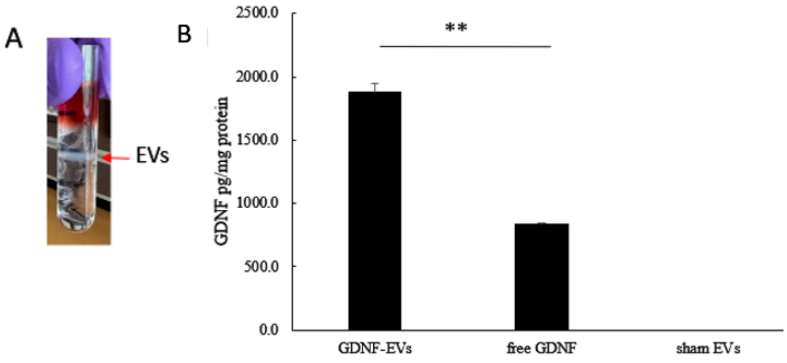


**Supplementary Figure S3. GDNF levels in EVs released by genetically modified macrophages by ELISA**. Bone-marrow derived macrophages (BMM) were transfected by electroporation using the optimal condition #4 (**Supplementary Figure S2**). Then, EVs were collected from conditioned media of GDNF-BMM for 24 hours and purified by a density gradient (**A**). GDNF levels in EVs fraction were significantly greater than those in non-EVs fraction (**B**). No GDNF was detected in EVs released by sham-transfected macrophages. Values are means ±SD, n = 4, $^{**}p < 0.005$; $^{***}p < 0.0005$.

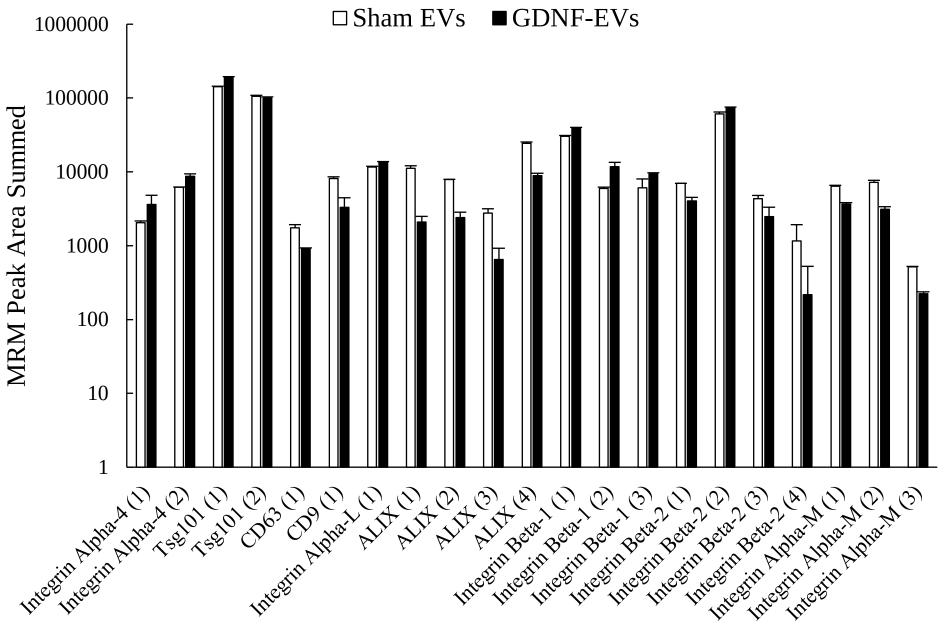


**Supplementary Figure S4. Quantification of Integrins and Tetraspanins in EVs by Label Free Targeted Quantitative Proteomics.** EVs samples from sham-transfected (white bars), and GDNF-transfected (black bars) macrophages were digested (n=3) with trypsin and examined by nano-liquid chromatography tandem MS (nanoLC–MS/MS) with multiple reaction monitoring (MRM). Samples of 20 µg total protein were used, and 0.08 µg (0.4 % of the sample) was injected. No significant differences in specific proteins expression were found between sham EVs and EV-GDNF (t-tests, $p < 0.05$). Peptide identification is shown in **Supplementary Table S3**. A CD81 peptide employed in other studies was not detected in these analyses. Values are means ± SD.

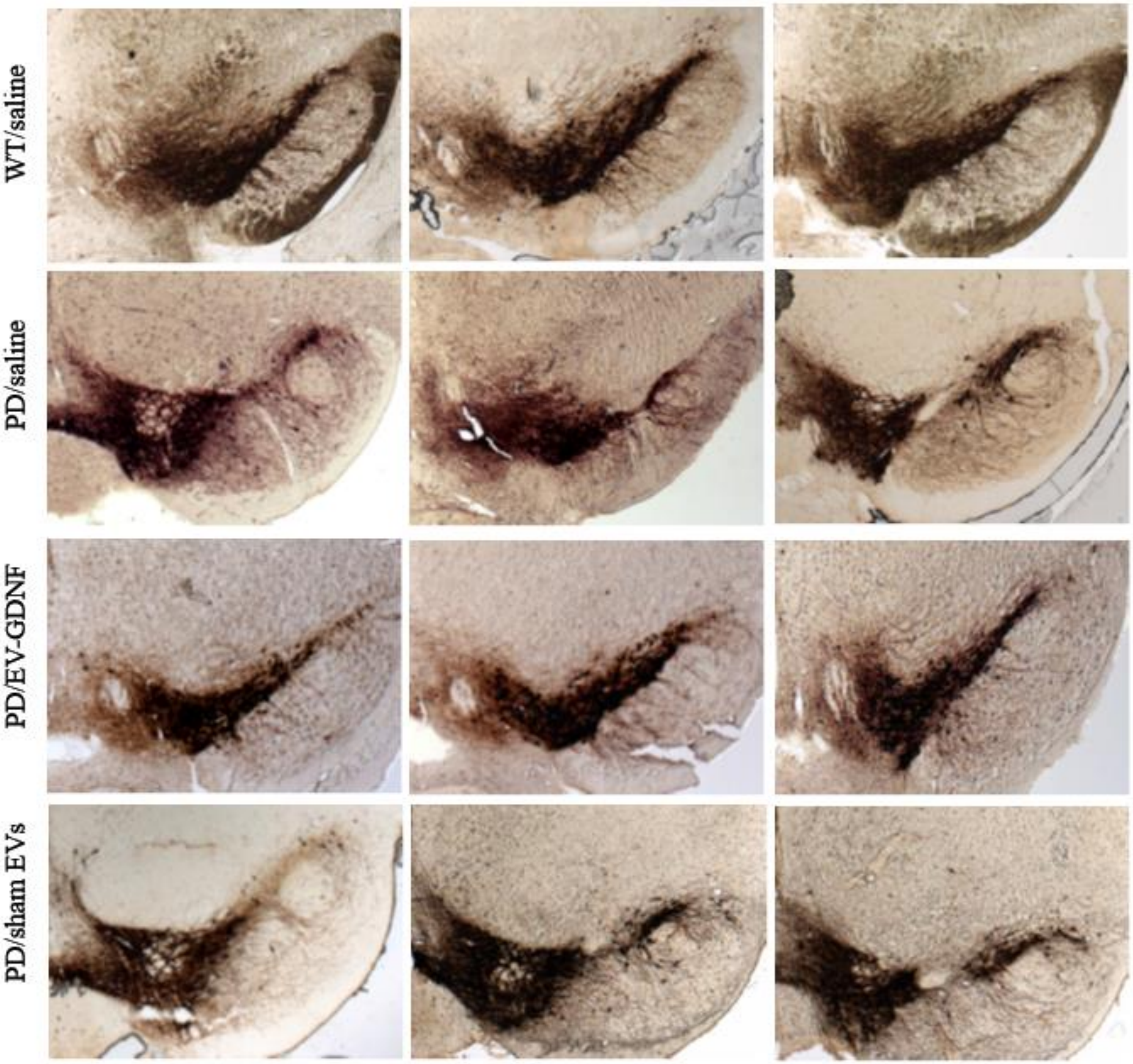


**Supplementary Figure S5. Neuroprotective effects of EV-GDNF in Parkin Q311(X)A mice.** Transgenic mice (4 mo. old, *n* = 10) were *i.n.* injected with: saline (10 µL/mouse), or EV-GDNF ($3x10^9$ particles/10 µL/mouse), or sham EVs ($3x10^9$ particles/10 µL/mouse). Wild type control mice (WT) were intranasally injected with saline (10 µL/mouse). Animals were sacrificed at mo. 16, and brain slides were stained with TH, a marker for dopaminergic neurons. The images indicate significant preservation of TH-positive neurons in Parkin Q311(X)A mice upon EV-GDNF treatment compared to PD mice treated with saline. The administration of sham EVs did not cause significant therapeutic effects.

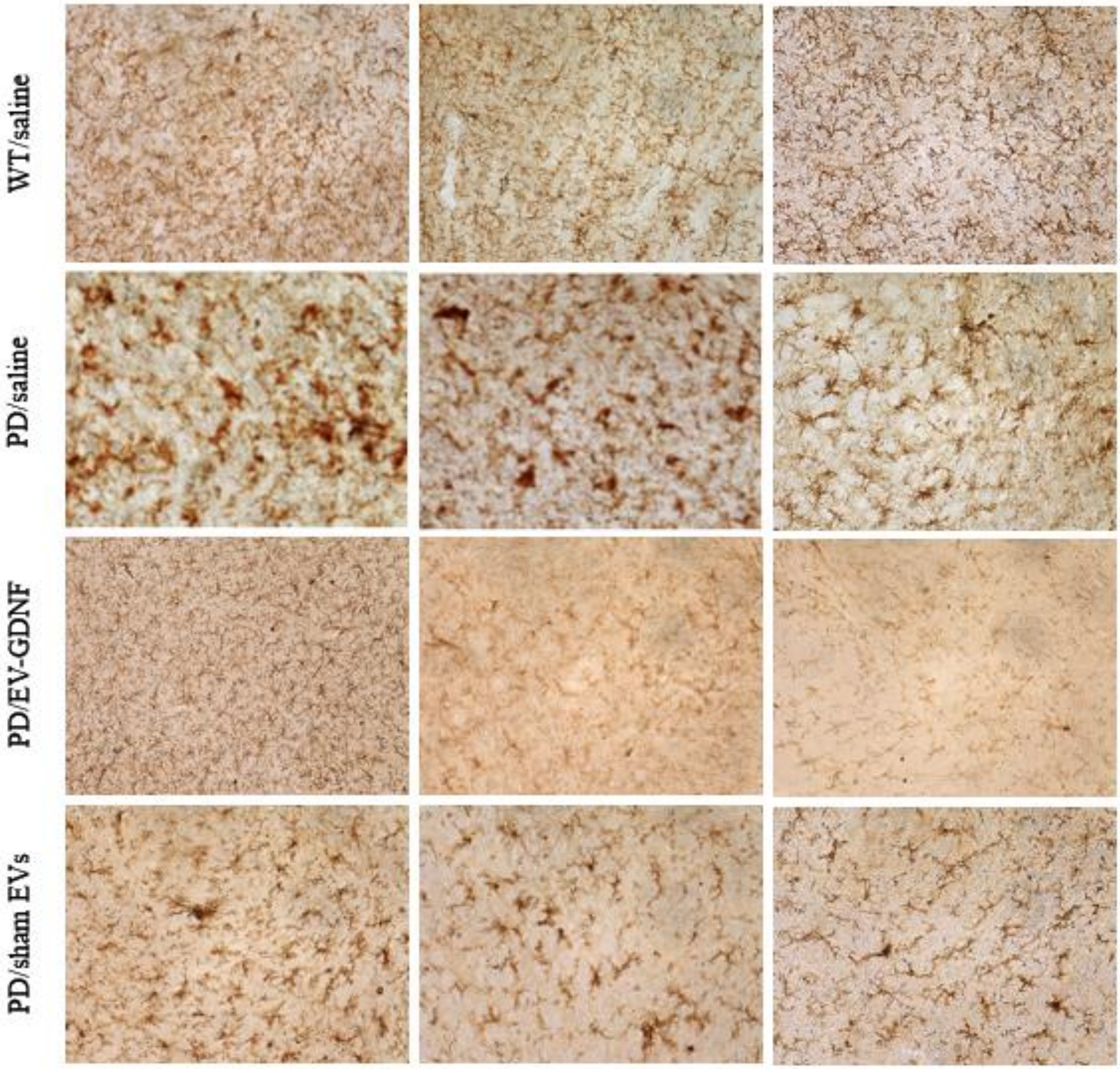


**Supplementary Figure S6. Anti-inflammatory effects of EV-GDNF in Parkin Q311(X)A mice.** Transgenic mice (4 mo. old, n = 10) were i.n. injected with: saline (10 µL/mouse), or EV-GDNF ($3x10^9$ particles/10 µL/mouse), or sham EVs (3x109 particles/10 µL/mouse). Wild type control mice (WT) were intranasally injected with saline (10 µL/mouse). Animals were sacrificed at mo. 16, and brain slides were stained with Ab to CD11b for activated microglia. The images indicate decrease in microglial activation in Parkin Q311(X)A mice upon EV-GDNF treatment compared to PD mice treated with saline. The administration of sham EVs did not cause significant therapeutic effects.

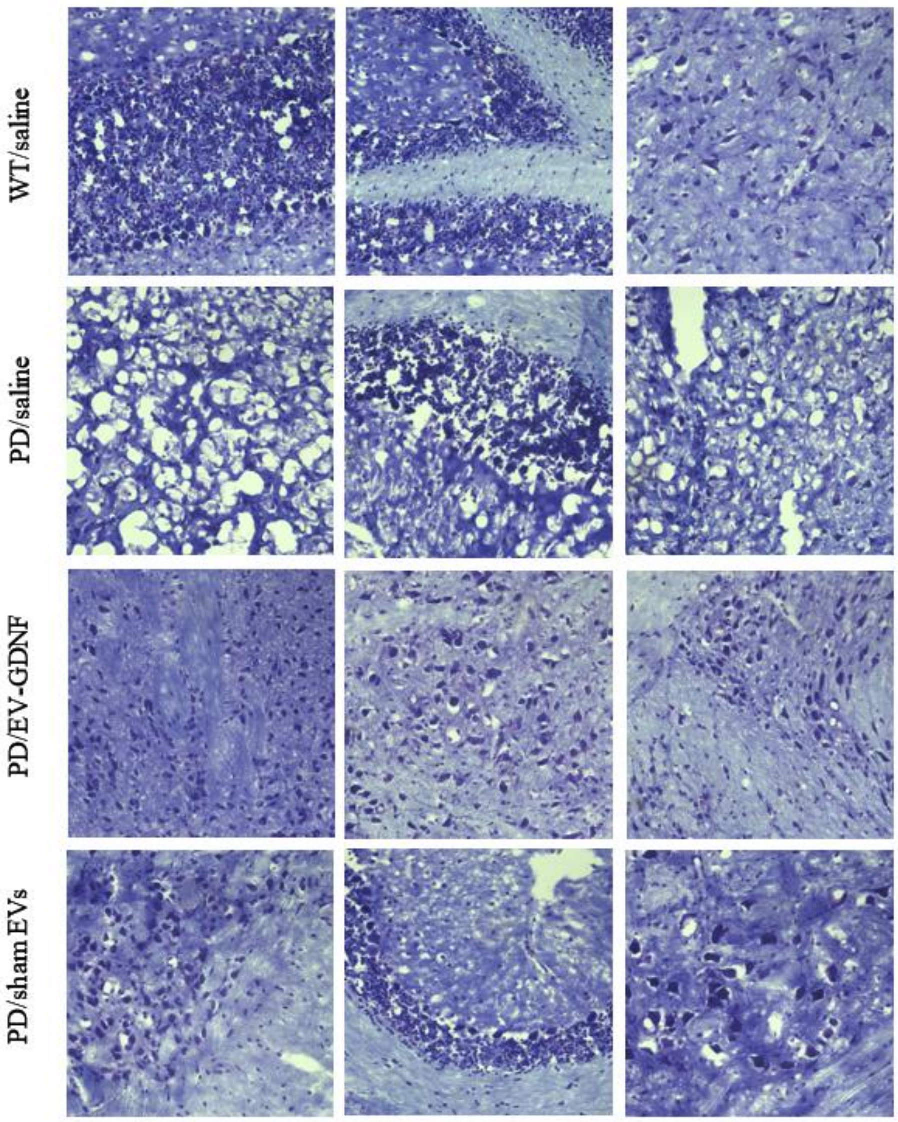


**Supplementary Figure S7. Histological analysis of neuroprotective effects by EV-GDNF in Parkin Q311(X)A mice.** Transgenic mice (4 mo. old) were intranasally injected with:  saline (10 µL/mouse), or EV-GDNF ($3x10^9$ particles/10 µL/mouse), or sham EVs ($3x10^9$ particles/10 µL/mouse) weekly three times. Wild type control mice were intranasally injected with saline (10 µL/mouse).  Animals were sacrificed at mo. 16, brain slides were stained with Nissl staining. The obtained bright light images show lower number of Nissl bodies with neuronal shrinkage and damages tissues with degeneration in the neurons in PD mice treated with saline when compared to WT mice. Histological analysis indicates neuroprotective effects in the brain of PD mice treated with GDNF-EVs with healthy morphology in tissue structure and high integrity of neurons when comparted to PD mice treated with saline. The administration of sham EVs did not have significant therapeutic effect in PD mice.

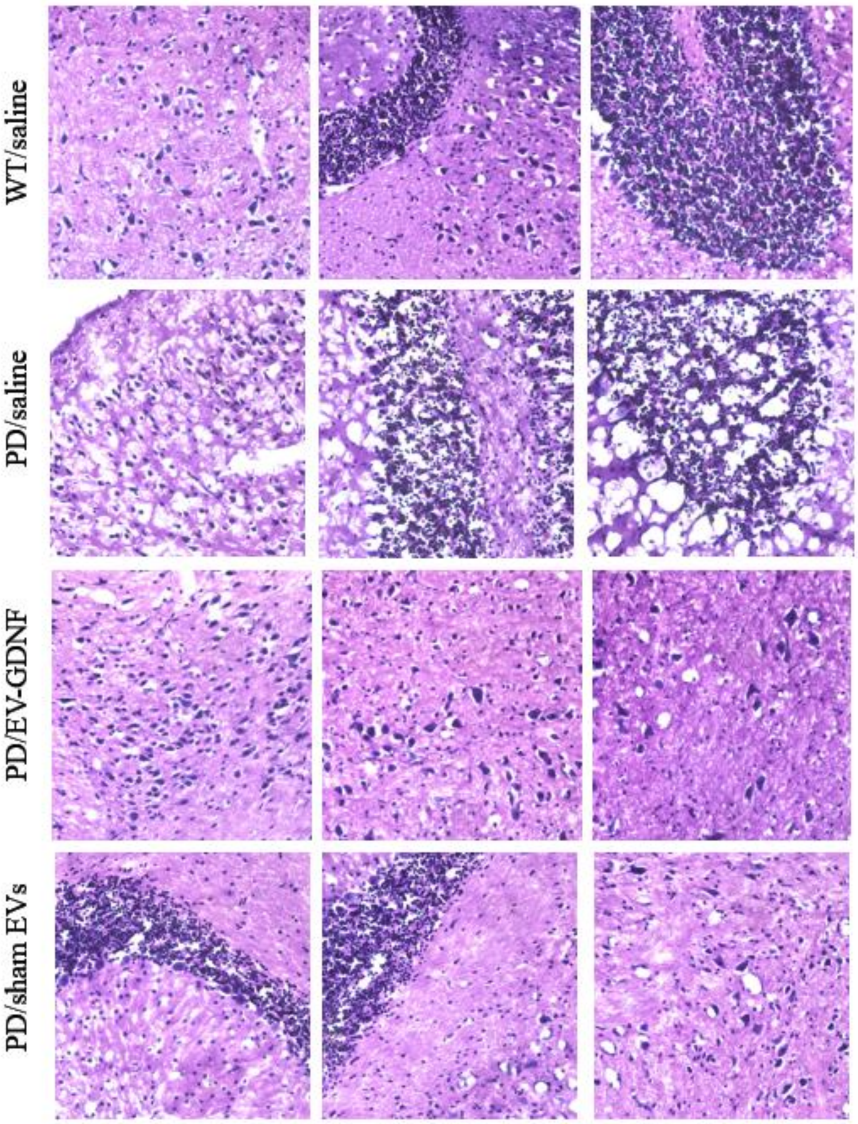


**Supplementary Figure S8. Histological analysis of neuroprotective effects by EV-GDNF in Parkin Q311(X)A mice.** Transgenic mice (4 mo. old) were intranasally injected with: saline (10 µL/mouse), or EV-GDNF ($3x10^9$ particles/10 µL/mouse), or sham EVs ($3x10^9$ particles/10 µL/mouse) weekly three times. Wild type control mice were intranasally injected with saline (10 µL/mouse). Animals were sacrificed at mo. 16, brain slides were stained with Nissl staining. The obtained bright light images show damaged tissues with degeneration in the neurons in PD mice treated with saline when compared to WT mice. Histological analysis indicates neuroprotective effects in the brain of PD mice treated with GDNF-EVs with healthy morphology in tissue structure when comparted to PD mice treated with saline. The administration of sham EVs did not have significant therapeutic effect in PD mice.

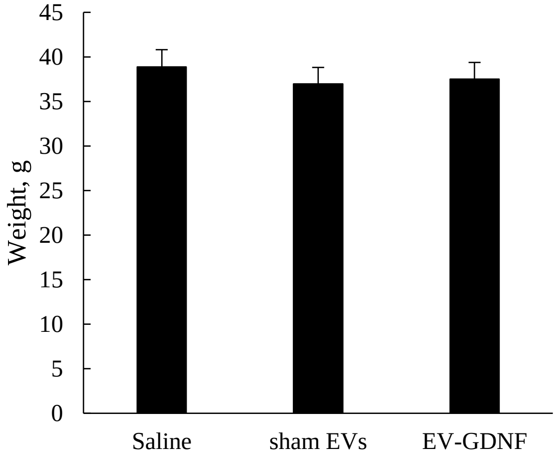


**Supplementary Figure S9. Absence of gross toxicity of EV-GDNF treatment in Parkin Q311(X)A mice**. Transgenic mice (4 mo. of age) were *i.n.* injected with saline, or EV-GDNF, or sham EVs ($3x10^9$ particles/10 µL/mouse) weekly three times. At 16 mo. of age total weigh of the animals was recorded. No gross toxicity manifested in the losing weight was detected in mice injected with EV-GDNF and well as sham EVs.

**Supplementary Table S1. Operation parameters of parent macrophages electroporation upon transfection with GDNF-encoding *p*DNA.**

| Conditions | Voltage, V | Width | Pulses |
|---|---|---|---|
| #1 | 0 | 1 | 1 |
| #2 | 1300 | 20 | 2 |
| #3 | 1700 | 20 | 2 |
| #4 | 1400 | 20 | 2 |

**Supplementary Table S2. Primary antibodies used for Simple Western Blot.**

| Antibody | Manufacturer | ID | Stock concentration mg/ml | Dilution factor | Protein concentration mg/ml |
|---|---|---|---|---|---|
| CD63 | Novus | NBP2-67425 | 1.000 | 100 | 40 |
| CD81 | Abcam | ab109201 | 0.229 | 10 | 200 |
| CD9 | Abcam | ab92726 | 0.111 | 10 | 200 |
| HSP90 beta | Novus | NBP2-67395 | 1.000 | 50 | 200 |
| TSG101 | Novus | NBP2-67884 | 1.000 | 500 | 40 |
| β Actin | Abcam | ab213262 | 0.500 | 50 | 200 |

**Supplementary Table S3. Murine integrin proteotypic tryptic peptides detectable for the label free quantitative assessment.**

| Protein | Peptide |
|---|---|
| Integrin Alpha-4.(1) | DNQWLGVTLSR |
| Integrin Alpha-4.(2) | QPGENGSIVTCGHR |
| Tsg101.(1) | DLKPVLDSYVFNDGSSR |
| Tsg101.(2) | ASLISAVSDK |
| CD63.(1) | TATILDK |
| CD9.(1) | ELQEFYK |
| Integrin Alpha-L.(1) | GHAVVGAVGAK |
| ALIX.(1) | GSLFGGSVK |
| ALIX.(2) | LALASLGYEK |
| ALIX.(3) | LANQAADYFGDAFK |
| ALIX.(4) | ELPELLQR |
| Integrin Beta-1.(1) | IGFGSFVEK |
| Integrin Beta-1.(2) | LLVFSTDAGFHFAGDGK |
| Integrin Beta-1.(3) | WDTGENPIYK |
| Integrin Beta-2.(1) | IGFGSFVDK |
| Integrin Beta-2.(2) | LGAILTPNDGR |
| Integrin Beta-2.(3) | SNEFDYPSVGQLAHK |
| Integrin Beta-2.(4) | SQWNNDNPLFK |
| Integrin Alpha-M.(1) | ILVVITDGEK |
| Integrin Alpha-M.(2) | VQSLVLGAPR |
| Integrin Alpha-M.(3) | GNLSFDWYIK |

**Supplementary Table S4. Effect of GDNF-EVs on neurodegeneration and neuroinflammation in Parkin Q311(X)A mice.**

| Treatment group | Effect | |
|---|---|---|
| | Total TH+ neurons x $10^3$ | Reactive Mac 1+, cell/mm2 |
| WT/saline | 7.3 ± 1.0 (*) | 9 ± 2.2 (*) |
| PD/EV-GDNF | 5.4 ± 1.4 (*) | 37 ± 5 (*) |
| PD/saline | 1.7 ± 0.2 | 101 ± 8 |
| PD/sham EVs | 2.5 ± 0.6 | 87 ± 7 |

[a] Total number of neurons was calculated in one hemisphere

[b] Statistical significance is shown by asterisk: *p*,0.05 (*) compared to PD mice with *i.n.* saline injections (PD controls). Errors are mean ± SEM, n = 10

**Supplementary Table S5. Effect of EV-GDNF on the expression of pro-inflammatory cytokines**

| Cytokine (pg/mL) | Liver | | Spleen | |
|---|---|---|---|---|
| | WT/saline | PD/EV-GDNF | WT/saline | PD/EV-GDNF |
| INF-γ | 6.5 ± 1.5 | 6.8 ± 1.2 | 5.9 ± 0.5 | 8.0 ± 0.2 |
| IP-10 | 146 ± 3 | 115 ± 11 | 156 ± 7 | 158 ± 4 |
| IL-4 | 24 ± 2 | 19 ± 1 | 20 ± 1 | 19 ± 2 |
| IL-6 | 6.5 ± 0.8 | 5.3 ± 0.4 | 9.0 ± 0.1 | 11.1 ± 3 |
| RANTES | 22.5 ± 3 | 12.5 ± 2.5 | 21 ± 3 | 15.2 ± 1 |
| MCP-1 | 5.1 ± 1.1 | 3.2 ± 0.7 | 2.9 ± 0.3 | 3.0 ± 0.5 |
| TNF-α | 7.5 ± 2 | 9.5 ± 1.4 | 1.3 ± 0.5 | 1.5 ± 0.4 |
